# Introducing the issue on “Differential use of CCR5 versus CXCR4 by HIV-1. Pathogenic, Translational and Clinical Open Questions”

**DOI:** 10.1186/1479-5876-9-S1-I1

**Published:** 2011-01-27

**Authors:** Guido Poli, Luigi Buonaguro

**Affiliations:** 1 AIDS Immunopathogenesis Unit, Division of Immunology, Transplantation and Infectious Diseases, San Raffaele Scientific Institute, Milano, Italy; 2 Vita-Salute San Raffaele University, School of Medicine, Milano, Italy; 3 Lab. of Molecular Biology and Viral Oncogenesis & AIDS Reference Center, Istituto Nazionale Tumori “Fond. G. Pascale”, Naples, Italy; 4 Institute of Human Virology, University of Maryland School of Medicine, Baltimore, MD, USA

## 

The discovery of CXCR4 and CCR5 in 1996 by several Authors as the two main mandatory receptors for HIV-1 entry into CD4+ cells will be recognized as a milestone of AIDS research. The present issue on “*Differential use of CCR5 versus CXCR4 by HIV-1. Pathogenic, Translational and Clinical Open Questions*” is intended to summarize the most update aspects on the biology of such HIV-1 coreceptors, including contributions by some of the most renown scientists in the field.

In less than 10 years, a new anti-HIV drug (Maraviroc) blocking CCR5 has been licensed for use in the treatment of infected individuals and new candidate vaccines and microbicides are under study, targeting the interface between gp120 Env and chemokines receptors. Deeper and broader appreciation of coreceptors in AIDS immunopathogenesis has been made possible, i.e. evaluating the occurrence and relevance of “natural” anti-CCR5 antibodies.

In these years, CCR5 has clearly emerged as the key co-receptor and the AIDS pandemic is essentially driven by viral strains exclusively exploiting this entry co-receptor named  "R5-virus" ("R5-related" disease).  On the contrary, CXCR4-using viruses are confined to the advanced immunodeficient stage of subtype B infection, accelerating disease progression. Qualitative and quantitative differences between CCR5 and CXCR4-using viruses (usually dualtropic R5X4) have been identified and a multiple “gatekeeper” hypothesis has been proposed. In particular, experimental data have been collected showing that binding and infection through CCR5 results in superior and differential signaling capacity, as well as viral replication and mother-to-child transmission.

Finally, discovery of the chemokine entry co-receptors has also pointed out one of the main limits of non-human primate models of HIV infection based on SIV strains, which exclusively use CXCR4 as coreceptor.

In summary, the present issue updates some of the most relevant lines of research revolving around the interaction between HIV and its host target cells, in which CCR5 and CXCR4 chemokine receptors play a key role and represent a focal point for HIV/AIDS pathogenetic studies as well as therapeutic and preventive intervention strategies.

## Competing interests

The authors declare no competing financial or other interests.

